# 3-Chloro-6-{4-[3-(trifluoro­meth­yl)phen­yl]piperazin-1-yl}pyridazine

**DOI:** 10.1107/S1600536810004137

**Published:** 2010-02-06

**Authors:** Hakan Arslan, Semra Utku, Kenneth I. Hardcastle, Mehtap Gökçe, Sheri Lense

**Affiliations:** a Department of Chemistry, Emory University, Atlanta, GA 30322, USA; b Department of Chemistry, Faculty of Arts and Science, Mersin University, Mersin, TR-33343, Turkey; c Department of Pharmaceutical Chemistry, Faculty of Pharmacy, Mersin University, Mersin, TR-33169, Turkey; d Department of Pharmaceutical Chemistry, Faculty of Pharmacy, Gazi University, Ankara, TR-06330, Turkey

## Abstract

The title compound, C_15_H_14_ClF_3_N_4_, was synthesized from 3,6-dichloro­pyridazine and 1-[3-(trifluoro­meth­yl)phen­yl]piper­azine. The piperazine ring is flanked by 3-chloro­pyridazine and 3-trifluoro­methyl­phenyl rings and adopts a chair conformation, whereas the 3-chloro­pyridazine and 3-trifluoro­methyl­phenyl rings are planar, with maximum deviations of 0.0069 (13) and 0.0133 (14) Å, respectively. The crystal structure is stabilized by weak inter­molecular C—H⋯N hydrogen-bond inter­actions.

## Related literature

For the synthesis and analgesic and anti-inflammatory activity of pyridazinone and pyridazine derivatives, see: Arslan *et al.* (2010 ▶); Giri & Mukhopadhyay (1998 ▶); Boissier *et al.* (1963 ▶); Gokce *et al.* (2001 ▶, 2004 ▶, 2005 ▶, 2009 ▶); Sahin *et al.* (2004 ▶); Dundar *et al.* (2007 ▶). For general background to pyrazolone derivatives, see: Amir *et al.* (2008 ▶); Banoglu *et al.* (2004 ▶). For puckering parameters, see: Cremer & Pople (1975 ▶).
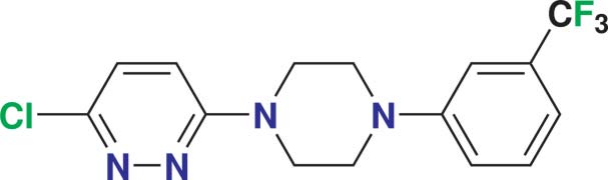


## Experimental

### 

#### Crystal data

C_15_H_14_ClF_3_N_4_
*M* *_r_* = 342.75
Monoclinic, 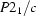

*a* = 9.461 (6) Å
*b* = 6.557 (4) Å
*c* = 24.123 (16) Å
β = 99.890 (9)°
*V* = 1474.1 (16) Å^3^
*Z* = 4
Mo *K*α radiation
μ = 0.30 mm^−1^
*T* = 173 K
0.41 × 0.25 × 0.24 mm

#### Data collection

Bruker APEXII CCD diffractometer
Absorption correction: multi-scan (*SADABS*; Bruker, 2008 ▶) *T*_min_ = 0.888, *T*_max_ = 0.932
19935 measured reflections
3385 independent reflections
2781 reflections with *I* > 2σ(*I*)
*R*_int_ = 0.065

#### Refinement

*R*[*F* ^2^ > 2σ(*F* ^2^)] = 0.036
*wR*(*F* ^2^) = 0.101
*S* = 1.06
3385 reflections
208 parameters
H-atom parameters constrained
Δρ_max_ = 0.32 e Å^−3^
Δρ_min_ = −0.23 e Å^−3^

### 

Data collection: *APEX2* (Bruker, 2008 ▶); cell refinement: *SAINT* (Bruker, 2008 ▶); data reduction: *SAINT*; program(s) used to solve structure: *SHELXS97* (Sheldrick, 2008 ▶); program(s) used to refine structure: *SHELXL97* (Sheldrick, 2008 ▶); molecular graphics: *SHELXTL* (Sheldrick, 2008 ▶); software used to prepare material for publication: *SHELXTL*.

## Supplementary Material

Crystal structure: contains datablocks I, global. DOI: 10.1107/S1600536810004137/hg2643sup1.cif

Structure factors: contains datablocks I. DOI: 10.1107/S1600536810004137/hg2643Isup2.hkl

Additional supplementary materials:  crystallographic information; 3D view; checkCIF report

## Figures and Tables

**Table 1:** Hydrogen-bond geometry (Å, °)

*D*—H⋯*A*	*D*—H	H⋯*A*	*D*⋯*A*	*D*—H⋯*A*
C11—H11*B*⋯N4^i^	0.99	2.69	3.628 (2)	158

Symmetry code: (i) 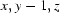.

**Fig. 1.**
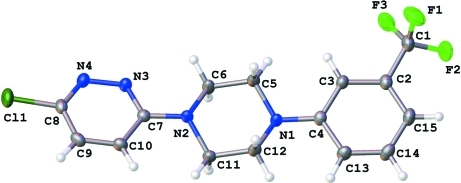
The molecular structure of (I), showing ellipsoids at the 50% probability level.

**Fig. 2.**
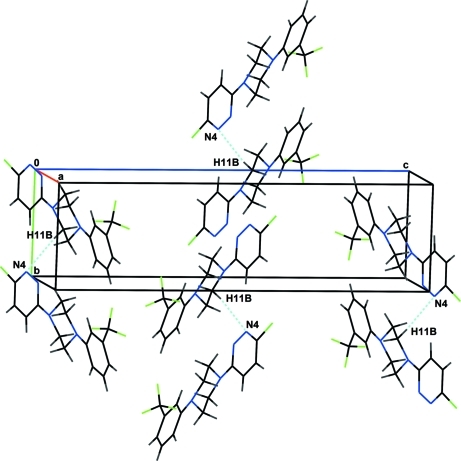
The molecular packing of (I). The hydrogen bonds are shown as dashed lines.

**Fig. 3.**
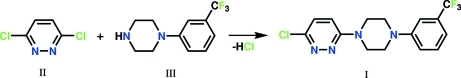
Synthesis of the title compound.

## supplementary crystallographic information

### Comment

It is known that some pyrazolone derivatives like oxyphenbutazone, dipyrone, antipyrine and phenylbutazone are used primarily for their anti-inflammatory, antipyretic and analgesic activities, but several side effects have limited the clinical use of these drugs such as some of pyrazolone derivatives are toxic and carcinogenicin animals, clastogenic in somatic and germ cells of male mice, and also weakly mutagenic in Salmonella strain TA100 in presence of rat liver homogenate. In addition, some of pyrazolone derivatives induce peptic ulcer, hypersensitivity reaction, hepatitis, nephritis and bone marrow suppression (Giri & Mukhopadhyay, 1998).

Pyridazinone derivatives are structurally related to pyrazolone derivatives (Gokce *et al.*, 2009). Many pyridazinone derivatives have been reported as analgesic and anti-inflammatory agents without gastrointestinal side effect (Amir *et al.*, 2008, Banoglu *et al.*, 2004, Gokce *et al.*, 2009). This is agreement with in our experience in the pyridazinone field. (Dundar *et al.*, 2007; Gokce *et al.*, 2001, 2004, 2005, 2009; Sahin *et al.*, 2004).

Recently, our research has focussed on the chemical, physical and biologycal properties of pyridazinone derivatives (Gokce *et al.*, 2009, Arslan *et al.*, 2010). The title compound, 3-chloro-6-{4-[3-(trifluoromethyl)phenyl]piperazin-1-yl}pyridazine, I, Scheme 1, is an example and in this article, we report on the crystal structure of the title compound, Figure 1.

The molecular structure of I consists of 3-chloropyridazine and 3-trifluoromethylphenyl arms connected to a piperazine ring. The 3-chloropyridazine and 3-trifluoromethylphenyl rings are planar with a maximum deviation of -0.0069 (13) Å for atom C7 and -0.0133 (14) Å for atom C3. The dihedral angle between these two rings is 18.77 (6) °. The piperazine ring adopts a chair conformation. This is confirmed by the puckering parameters *q*_2_ = 0.0107 (14) Å, *q*_3_= 0.5479 (13) Å, *Q*_T_ = 0.5480 (13) Å, *θ* = 1.05 (15) ° and φ= 85 (7) ° (Cremer & Pople, 1975).

The conformations of the 3-chloropyridazine and 3-trifluoromethylphenyl rings are best described by the torsion angles of 159.40 (11) ° and -165.62 (11) ° for C7—N2—C6—C5 and C4—N1—C12—C11, respectively; thus they adopt + antiperiplanar and - antiperiplanar conformations, respectively.

The crystal packing is dominated by weak intermolecular C11—H11B···N4 (*x*, *y*-1, *z*) hydrogen bonds, with H···N = 2.69 Å and a C—H···N angle of 150 ° (Figure 2).

### Experimental

A mixture of 3,6-dichloropyridazine, II, (1.7 mol) and 1-[3-(trifluoromethyl)phenyl]piperazine, III, (2.0 mol) in ethanol (10 ml) was heated under reflux for 4 hours after which the mixture was cooled to room temperature (Figure 3) (Boissier *et al.* (1963)). The resulting crude precipitate was filtered off and purified by repeated washing with small portions of cold ethanol. The precipitate formed was crystallized from CH_2_Cl_2_: ethanol (5:10 ml) to give the compound, 3-chloro-6-{4-[3-(trifluoromethyl)phenyl]piperazin-1-yl}pyridazine, I, as white crystals. Yields: 0.485 g, 83%. M.p.: 167 °C. ^1^H-NMR (DMSO-d_6_) δ: 7.56-7.54 (d, 1H, pyridazin), 7.46-7.40 (m, 2H, phenyl), 7.28-7.21 (m, 1H, phenyl), 7.14-7.12 (d, 1H, phenyl), 7.09-7.07 (d, 1H, pyridazin), 3.74-3.71 (t, 2H, piperazine), 3.45-3.43 (t, 2H, piperazine), 3.19-3.17 (t, 4H, piperazine). MS (EI) m/z: 343 (M^+^). Anal. Calc. for C_15_H_14_N_4_ClF_3_: C, 52.56; H, 4.12; N, 16.35%. Found: C, 52.61; H, 4.09; N, 16.40%.

### Refinement

The H atoms were positioned geometrically and allowed to ride on their parent atoms, with C—H distances of 0.95 Å (CH) or 0.99 Å (CH_2_), and with *U*_iso_(H) = 1.2*U*_eq_ of the parent atoms.

### Crystal data

**Table:**

C_15_H_14_ClF_3_N_4_	*F*(000) = 704
*M**_r_* = 342.75	*D*_x_ = 1.544 Mg m^−^^3^
Monoclinic, *P*2_1_/*c*	Mo *K*α radiation, λ = 0.71073 Å
Hall symbol: -P 2ybc	Cell parameters from 7857 reflections
*a* = 9.461 (6) Å	θ = 2.2–29.7°
*b* = 6.557 (4) Å	µ = 0.30 mm^−^^1^
*c* = 24.123 (16) Å	*T* = 173 K
β = 99.890 (9)°	Block, colourless
*V* = 1474.1 (16) Å^3^	0.41 × 0.25 × 0.24 mm
*Z* = 4	

### Data collection

**Table:**

Bruker APEXII CCD diffractometer	3385 independent reflections
Radiation source: fine-focus sealed tube	2781 reflections with *I* > 2σ(*I*)
graphite	*R*_int_ = 0.065
φ and ω scans	θ_max_ = 27.5°, θ_min_ = 1.7°
Absorption correction: multi-scan (*SADABS*; Bruker, 2008)	*h* = −12→12
*T*_min_ = 0.888, *T*_max_ = 0.932	*k* = −8→8
19935 measured reflections	*l* = −31→31

### Refinement

**Table:**

Refinement on *F*^2^	Primary atom site location: structure-invariant direct methods
Least-squares matrix: full	Secondary atom site location: difference Fourier map
*R*[*F*^2^ > 2σ(*F*^2^)] = 0.036	Hydrogen site location: inferred from neighbouring sites
*wR*(*F*^2^) = 0.101	H-atom parameters constrained
*S* = 1.06	*w* = 1/[σ^2^(*F*_o_^2^) + (0.0589*P*)^2^ + 0.1216*P*] where *P* = (*F*_o_^2^ + 2*F*_c_^2^)/3
3385 reflections	(Δ/σ)_max_ = 0.005
208 parameters	Δρ_max_ = 0.32 e Å^−^^3^
0 restraints	Δρ_min_ = −0.23 e Å^−^^3^

### Special details

**Table:**

Geometry. All esds (except the esd in the dihedral angle between two l.s. planes) are estimated using the full covariance matrix. The cell esds are taken into account individually in the estimation of esds in distances, angles and torsion angles; correlations between esds in cell parameters are only used when they are defined by crystal symmetry. An approximate (isotropic) treatment of cell esds is used for estimating esds involving l.s. planes.
Refinement. Refinement of *F*^2^ against ALL reflections. The weighted *R*-factor wR and goodness of fit *S* are based on *F*^2^, conventional *R*-factors *R* are based on *F*, with *F* set to zero for negative *F*^2^. The threshold expression of *F*^2^ > σ(*F*^2^) is used only for calculating *R*-factors(gt) etc. and is not relevant to the choice of reflections for refinement. *R*-factors based on *F*^2^ are statistically about twice as large as those based on *F*, and *R*- factors based on ALL data will be even larger.

### Fractional atomic coordinates and isotropic or equivalent isotropic displacement parameters (Å^2^)

**Table:**

	*x*	*y*	*z*	*U*_iso_*/*U*_eq_	
C1	1.35124 (16)	0.4693 (2)	0.76675 (6)	0.0301 (3)	
C2	1.23214 (15)	0.35744 (19)	0.78778 (5)	0.0228 (3)	
C3	1.15571 (14)	0.45817 (19)	0.82401 (5)	0.0212 (3)	
H3	1.1811	0.5939	0.8354	0.025*	
C4	1.04104 (14)	0.36134 (18)	0.84400 (5)	0.0194 (3)	
C5	1.01095 (15)	0.65546 (17)	0.90409 (6)	0.0235 (3)	
H5A	1.0563	0.7374	0.8775	0.028*	
H5B	1.0856	0.6219	0.9368	0.028*	
C6	0.89367 (15)	0.78043 (18)	0.92372 (6)	0.0241 (3)	
H6A	0.9364	0.9031	0.9439	0.029*	
H6B	0.8240	0.8262	0.8906	0.029*	
C7	0.74380 (13)	0.76537 (18)	0.99646 (5)	0.0186 (3)	
C8	0.62135 (14)	0.9883 (2)	1.06758 (5)	0.0220 (3)	
C9	0.60062 (14)	0.7770 (2)	1.06879 (5)	0.0236 (3)	
H6	0.5458	0.7163	1.0939	0.028*	
C10	0.66253 (14)	0.66193 (19)	1.03229 (5)	0.0221 (3)	
H10	0.6517	0.5179	1.0309	0.027*	
C11	0.76124 (14)	0.47012 (18)	0.93414 (6)	0.0223 (3)	
H11A	0.6846	0.5022	0.9019	0.027*	
H11B	0.7183	0.3881	0.9614	0.027*	
C12	0.87797 (15)	0.34660 (18)	0.91356 (6)	0.0226 (3)	
H12A	0.9476	0.2987	0.9464	0.027*	
H12B	0.8344	0.2250	0.8930	0.027*	
C13	1.01216 (15)	0.15733 (18)	0.82706 (5)	0.0238 (3)	
H13	0.9365	0.0862	0.8399	0.029*	
C14	1.09205 (16)	0.05852 (19)	0.79199 (5)	0.0277 (3)	
H14	1.0708	−0.0796	0.7819	0.033*	
C15	1.20215 (16)	0.1564 (2)	0.77128 (6)	0.0268 (3)	
H15	1.2552	0.0886	0.7467	0.032*	
Cl1	0.54631 (4)	1.14481 (6)	1.113190 (14)	0.03333 (13)	
F1	1.47712 (10)	0.45600 (17)	0.80245 (4)	0.0510 (3)	
F2	1.37526 (11)	0.39838 (16)	0.71726 (4)	0.0517 (3)	
F3	1.32478 (10)	0.66983 (13)	0.75974 (4)	0.0438 (3)	
N1	0.95396 (11)	0.46588 (15)	0.87639 (4)	0.0201 (2)	
N2	0.81888 (12)	0.66056 (15)	0.96101 (4)	0.0201 (2)	
N3	0.75776 (12)	0.96974 (15)	0.99729 (4)	0.0233 (3)	
N4	0.69511 (12)	1.08204 (16)	1.03382 (5)	0.0248 (3)	

### Atomic displacement parameters (Å^2^)

**Table:**

	*U*^11^	*U*^22^	*U*^33^	*U*^12^	*U*^13^	*U*^23^
C1	0.0290 (8)	0.0347 (7)	0.0294 (7)	−0.0006 (6)	0.0132 (6)	−0.0075 (6)
C2	0.0230 (7)	0.0259 (6)	0.0203 (6)	0.0020 (5)	0.0054 (5)	−0.0013 (5)
C3	0.0239 (7)	0.0189 (6)	0.0219 (6)	−0.0001 (5)	0.0065 (5)	−0.0028 (5)
C4	0.0236 (7)	0.0184 (6)	0.0163 (6)	0.0026 (5)	0.0040 (5)	0.0014 (4)
C5	0.0269 (7)	0.0156 (6)	0.0314 (7)	−0.0049 (5)	0.0147 (6)	−0.0026 (5)
C6	0.0318 (8)	0.0149 (5)	0.0298 (7)	−0.0027 (5)	0.0175 (6)	0.0001 (5)
C7	0.0176 (7)	0.0194 (6)	0.0189 (6)	−0.0007 (5)	0.0032 (5)	0.0011 (4)
C8	0.0196 (7)	0.0285 (6)	0.0181 (6)	0.0016 (5)	0.0038 (5)	−0.0036 (5)
C9	0.0201 (7)	0.0307 (7)	0.0211 (6)	−0.0022 (5)	0.0063 (5)	0.0041 (5)
C10	0.0225 (7)	0.0202 (6)	0.0244 (6)	−0.0025 (5)	0.0063 (5)	0.0031 (5)
C11	0.0231 (7)	0.0182 (6)	0.0274 (7)	−0.0055 (5)	0.0094 (5)	−0.0023 (5)
C12	0.0281 (8)	0.0144 (5)	0.0278 (7)	−0.0037 (5)	0.0118 (6)	−0.0007 (5)
C13	0.0314 (8)	0.0186 (6)	0.0226 (6)	−0.0027 (5)	0.0081 (6)	0.0012 (5)
C14	0.0415 (9)	0.0184 (6)	0.0237 (6)	0.0011 (6)	0.0073 (6)	−0.0026 (5)
C15	0.0324 (8)	0.0258 (7)	0.0237 (7)	0.0055 (5)	0.0089 (6)	−0.0042 (5)
Cl1	0.0347 (2)	0.0404 (2)	0.0277 (2)	0.00403 (15)	0.01307 (16)	−0.00988 (14)
F1	0.0271 (6)	0.0704 (7)	0.0556 (6)	−0.0063 (5)	0.0077 (5)	0.0012 (5)
F2	0.0615 (7)	0.0616 (6)	0.0419 (6)	−0.0158 (5)	0.0369 (5)	−0.0197 (5)
F3	0.0458 (6)	0.0321 (5)	0.0617 (6)	−0.0037 (4)	0.0324 (5)	0.0035 (4)
N1	0.0250 (6)	0.0148 (5)	0.0228 (5)	−0.0022 (4)	0.0107 (5)	−0.0010 (4)
N2	0.0239 (6)	0.0144 (5)	0.0246 (6)	−0.0032 (4)	0.0115 (5)	0.0001 (4)
N3	0.0279 (7)	0.0186 (5)	0.0261 (6)	−0.0026 (4)	0.0121 (5)	−0.0027 (4)
N4	0.0279 (7)	0.0225 (5)	0.0258 (6)	−0.0017 (5)	0.0096 (5)	−0.0057 (4)

### Geometric parameters (Å, °)

**Table:**

C1—F2	1.3364 (16)	C8—N4	1.3129 (17)
C1—F3	1.3439 (18)	C8—C9	1.400 (2)
C1—F1	1.3472 (18)	C8—Cl1	1.7425 (14)
C1—C2	1.504 (2)	C9—C10	1.3654 (18)
C2—C15	1.3920 (19)	C9—H6	0.9500
C2—C3	1.3928 (18)	C10—H10	0.9500
C3—C4	1.4114 (18)	C11—N2	1.4681 (17)
C3—H3	0.9500	C11—C12	1.5199 (18)
C4—N1	1.4073 (16)	C11—H11A	0.9900
C4—C13	1.4115 (18)	C11—H11B	0.9900
C5—N1	1.4691 (17)	C12—N1	1.4676 (16)
C5—C6	1.5190 (18)	C12—H12A	0.9900
C5—H5A	0.9900	C12—H12B	0.9900
C5—H5B	0.9900	C13—C14	1.3878 (19)
C6—N2	1.4650 (16)	C13—H13	0.9500
C6—H6A	0.9900	C14—C15	1.388 (2)
C6—H6B	0.9900	C14—H14	0.9500
C7—N3	1.3462 (17)	C15—H15	0.9500
C7—N2	1.3845 (16)	N3—N4	1.3600 (15)
C7—C10	1.4236 (17)		
			
F2—C1—F3	106.53 (12)	C10—C9—H6	121.4
F2—C1—F1	106.34 (12)	C8—C9—H6	121.4
F3—C1—F1	105.59 (12)	C9—C10—C7	117.68 (12)
F2—C1—C2	112.59 (12)	C9—C10—H10	121.2
F3—C1—C2	112.66 (11)	C7—C10—H10	121.2
F1—C1—C2	112.60 (13)	N2—C11—C12	111.21 (11)
C15—C2—C3	121.75 (12)	N2—C11—H11A	109.4
C15—C2—C1	119.53 (12)	C12—C11—H11A	109.4
C3—C2—C1	118.72 (12)	N2—C11—H11B	109.4
C2—C3—C4	120.89 (12)	C12—C11—H11B	109.4
C2—C3—H3	119.6	H11A—C11—H11B	108.0
C4—C3—H3	119.6	N1—C12—C11	112.04 (11)
N1—C4—C13	121.30 (11)	N1—C12—H12A	109.2
N1—C4—C3	121.89 (11)	C11—C12—H12A	109.2
C13—C4—C3	116.70 (11)	N1—C12—H12B	109.2
N1—C5—C6	111.56 (11)	C11—C12—H12B	109.2
N1—C5—H5A	109.3	H12A—C12—H12B	107.9
C6—C5—H5A	109.3	C14—C13—C4	121.34 (12)
N1—C5—H5B	109.3	C14—C13—H13	119.3
C6—C5—H5B	109.3	C4—C13—H13	119.3
H5A—C5—H5B	108.0	C15—C14—C13	121.65 (12)
N2—C6—C5	110.97 (11)	C15—C14—H14	119.2
N2—C6—H6A	109.4	C13—C14—H14	119.2
C5—C6—H6A	109.4	C14—C15—C2	117.62 (12)
N2—C6—H6B	109.4	C14—C15—H15	121.2
C5—C6—H6B	109.4	C2—C15—H15	121.2
H6A—C6—H6B	108.0	C4—N1—C12	118.34 (10)
N3—C7—N2	116.34 (10)	C4—N1—C5	117.42 (11)
N3—C7—C10	121.81 (11)	C12—N1—C5	110.68 (10)
N2—C7—C10	121.77 (12)	C7—N2—C6	117.79 (10)
N4—C8—C9	124.58 (11)	C7—N2—C11	120.25 (11)
N4—C8—Cl1	115.68 (10)	C6—N2—C11	111.53 (10)
C9—C8—Cl1	119.73 (10)	C7—N3—N4	119.73 (10)
C10—C9—C8	117.13 (12)	C8—N4—N3	119.05 (11)

### Hydrogen-bond geometry (Å, °)

**Table:**

*D*—H···*A*	*D*—H	H···*A*	*D*···*A*	*D*—H···*A*
C11—H11B···N4^i^	0.99	2.69	3.628 (2)	158

Symmetry codes: (i) *x*, *y*−1, *z*.
